# Molecular phylogeny and taxonomy of the *Epictia goudotii* Species complex (Serpentes: Leptotyphlopidae: Epictinae) in Middle America and northern South America

**DOI:** 10.7717/peerj.1551

**Published:** 2016-01-12

**Authors:** James R. McCranie, S. Blair Hedges

**Affiliations:** 1 Smithsonian Institute, Miami, Florida, USA; 2 Center for Biodiversity, Temple University, USA

**Keywords:** *Epictia albifrons*, *Epictia tenella*, Middle America, *Epictia goudotii* Species complex, Northern South America, Threadsnakes, Molecular phylogeny

## Abstract

Here we review the systematics of the threadsnakes of the *Epictia goudotii* Species complex in Middle and northern South America using external morphology and molecular data. Two species, *Epictia goudotii* and *E. magnamaculata*, are currently recognized from that region, but we provide evidence for recognizing, as species, three other nominal forms usually treated as subspecies of *E. goudotii*: *E. ater, E. bakewelli*, and *E. phenops*. Thus, together with *E. columbi* (Bahamas), we recognize six species in the *Epictia goudotii* Species complex. Because *E. albifrons* from northern South America has been confused with *E. goudotii* in the past, we also briefly discuss the taxonomic status of that species and its apparent close relative *E. tenella*, which are not members of the *E. goudotii* complex.

## Introduction

The Genus *Epictia*, Gray (1845) contains approximately 25 species of threadsnakes occurring in the New World, although nearly all of them are restricted to South America (Adalsteinsson et al., 2009). One wide-ranging species, *Epictia goudotii* (Duméril & Bibron, 1844; as *Stenostoma* Wagler, 1824), occurs in northern South America and Middle America and has had a long taxonomic history during which some populations have been recognized as either subspecies or species. Adalsteinsson et al. (2009) provided molecular evidence that *Epictia goudotii* is actually a complex of species and elevated one previously recognized subspecies to a species (*E. magnamaculata* (Taylor, 1940; as *Leptotyphlops*)). Those results of Adalsteinsson et al. (2009), along with those of McCranie (2011) noting that some of the morphological variation reported in the literature for *E. goudotii* was also associated with geography, prompted this current study. Herein, we gather new molecular and morphological evidence that justifies the recognition of additional species of snakes in this Species complex.

The nomenclatural and taxonomic histories, both at the generic and specific levels, of the Mexican to South American members of the *Epictia goudotii* Species complex have suffered from considerable confusion in the literature. Members of this complex were usually previously placed in *Stenostoma* until that name was found to be preoccupied (see Peters & Orejas-Miranda, 1970). Boulenger (1893) used *Glauconia* Gray (1845) for those members of *Epictia*. Barbour (1914) placed the Honduran Bay Island populations of this complex in *Leptotyphlops* Fitzinger (1843), where they usually remained until Adalsteinsson et al. (2009) erected *Epictia* for 25 species, including *E. goudotii* and *E. albifrons* (Wagler, 1824; as *Stenostoma*). *Epictia albifrons* is usually associated with the *E. goudotii* complex or *E. goudotii* with the *E. albifrons* group (see Peters & Orejas-Miranda, 1970).

Adalsteinsson et al. (2009) included two genetic samples (ROM 20503, 22487), they identified as *E. albifrons* from Guyana, and found that they were as different genetically from *E. goudotii* as were different genera elsewhere in their tree. This indicates that *E. goudotii* and the species studied herein belong to a different species group than do *E. albifrons* and *E. tenella*. Wagler (1824) described *Stenostoma albifrons* based on one specimen from “in adjacentibus Urbis Para (= in the proximity of Pará, Brazil)” (p. 69), although those locality data could be erroneous (see Wallach, Williams & Boundy, 2014). Smith & List (1958) noted that Wagler’s (1824) description and illustration of *S. albifrons* were inadequate and could apply to a large number of threadsnakes with “a light spot on head and tail.” Smith & List (1958) also discovered that the type specimen of *S. albifrons* was destroyed during World War II prompting those authors to say that a neotype from the type locality “should be designated and redescribed.” Orejas-Miranda (1967) was not able to find any museum specimens he could assign to *E. albifrons* and noted that specimens he examined from the vicinity of its purported type locality were in reality *E. tenella* (Klauber 1939; as *Leptotyphlops* from “Kartabo, British Guiana”). Those results and the destroyed holotype led Wilson & Hahn (1973) to suggest that the name *Stenostoma albifrons* be designated a *nomen dubium* (Wilson & Hahn stated that Orejas-Miranda “chose not to designate a neotype” (p. 120) but Orejas-Miranda actually said that he could not find additional specimens of *E. albifrons* in any museum). Hoogmoed & Gruber (1983) suggested placing *E. tenella* in the synonymy of *E. albifrons* in an effort to provide a workable definition of *E. albifrons*, and this was done by Wallach (In: McDiarmid, Campbell & Touré, 1999). Franco & Pinto (2010) considered *E. albifrons* a *nomen dubium*. More recently, Wallach, Williams & Boundy (2014) recognized both *E. albifrons* and *E. tenella* as valid species, but gave no data to support that claim. Mumaw, González & Fernández (2015:289) designated a neotype for *E. albifrons* (MCZ R-2885) from the vicinity of “Pará, Brasil” and provided a description of the specimen. The taxonomic status (*E. albifrons* vs. *E. tenella*) of the Guyana specimens sequenced by (Adalsteinsson et al. (2009); as *E. albifrons*) remain unsettled, but those sequence data are also included in this current study. An extensive revision of all *Epictia* species is needed, but because of the unavailable nature of genetic data from most South American populations (at least to North American researchers), and the few specimens of those populations in US museums, we focus herein on Mexican and Central American populations. All of the populations studied herein have usually been considered as subspecies of *E. goudotii* in previous literature (see Peters & Orejas-Miranda, 1970; as *Leptotyphlops*).

Available names associated with the Middle American forms of this complex, according to Dunn & Saxe (1950), include: *Epictia albifrons goudotii* from Panama and northwestern South America; *E. a. phenops* (*Stenostoma phenops* Cope, 1875; from southern Mexico and Guatemala); *E. a. bakewelli* (*Leptotyphlops bakewelli* Oliver, 1937; from Colima, Mexico); *E. a. magnamaculata* from Utila Island, Honduras); *E. nasalis* (*Leptotyphlops nasalis* Taylor, 1940; from Managua, Nicaragua); and *E. ater* (*Leptotyphlops ater* Taylor, 1940; from Managua, Nicaragua). Dunn & Saxe (1950), as first revisers, considered *E. nasalis* and *E. ater* synonymous and chose *E. ater* over *E. nasalis* as the name for this taxon. Duellman (1956) described *Leptotyphlops gadowi* from Michoacán, Mexico, but that name has rarely been mentioned in the literature and was listed in the synonymy of *E. goudotii phenops* by Hahn (1980) and in the synonymy of *E. bakewelli* by Wallach, Williams & Boundy (2014:276).

Peters & Orejas-Miranda (1970) recognized five subspecies of *Epictia goudotii* and were followed by most workers until Savage (2002), in using color comments by Peters & Orejas-Miranda (1970), noted that slight color differences existed among the Costa Rican and Nicaraguan populations and those of the northern South American members this complex usually assigned to *E. g. goudotii*. Savage (2002) also noted that the Costa Rican and Nicaraguan populations have the rostral and frontal (as prefrontal) scale fused (= frontal scale absent), whereas those from northern South America lack the rostral-frontal fusion (= frontal scale present, see Materials and Methods) and that both populations are completely allopatric to each other. Thus, Savage (2002) considered the Costa Rican and Nicaraguan populations to be *E. ater* and those of northern South America *E. goudotti* (sic).

McCranie (2011) included the Honduran mainland populations of this complex as *E. ater*, largely following Savage, and those from the Honduran Caribbean islands to be *E. magnamaculata* based on morphological data plus the genetic results of Adalsteinsson et al. (2009). McCranie (2011) also discussed the possibility that both *E. phenops* and *E. bakewelli* were valid species as well, based on the presence or absence of rostral-frontal fusion and some color pattern differences. Wallach, Williams & Boundy (2014) considered both *E. bakewelli* and *E. phenops* as valid species, but without providing supporting data.

In this report on the *Epictia goudotii* complex, we use genetic and external morphological data in an effort to resolve the systematics of the available Mexican (largely Pacific versant populations), Central American, and northern coastal South American populations. However, we stress that the taxonomic confusion surrounding *E. albifrons* and *E. tenella* (briefly discussed herein) is a serious obstacle in understanding the systematics and correct taxonomy of most northern South American populations. This is true despite the genetic evidence that “*E. albifrons* and *E. tenella*” from Guyana is apparently not closely related to the *E. goudotii* complex populations from Mexico and Central America (Adalsteinsson et al., 2009).

## Materials and Methods

The populations of *Epictia goudotii* under study herein are those occurring on the mainland from Mexico to northern South America, and those on the Islas de la Bahía and Swan Islands of Honduras. We also include the Bahamian species *E. columbi* (Klauber, 1939) shown to be closely related to *E. magnamaculata* in a previous molecular analysis (Adalsteinsson et al., 2009). Because of the unavailability of genetic material from much of South America, we have focused primarily on populations in Middle America (= Mexico and Central America) for this current study. The type locality of *E. goudotii* lies in Colombia, but unfortunately Colombian authorities do not allow exportation of tissued material from that country. Specimens were collected and exported by JRM with permission of the Honduras government, under permits Resolución DE-MP-102–2012 and Constancia 036–2012-DVS-ICF, Constancia 038–2012-DVS-ICF.

The molecular data set comprised sequences of two mitochondrial genes (12S rRNA and cytochrome b) from eight individuals of *Epictia* collected in Honduras and Mexico (Specimens Examined). These new sequences were aligned with GenBank sequences from 15 samples of *Epictia* from the West Indies, mainland Central America, and South America (see Adalsteinsson et al., 2009; *Epictia albifrons*/*E. tenella* 1—GQ469223, GQ469096; *Epictia albifrons*/*E. tenella* 2—GQ469224, GQ469097; *Epictia bakewelli* 1—GQ469221, GQ469122; *Epictia bakewelli* 2—GQ469220, GQ469121; *Epictia bakewelli* 4—GQ469217, GQ469123; *Epictia columbi* 1—GQ469213, GQ469091; *Epictia columbi* 2—GQ469212, GQ469089; *Epictia columbi* 3—GQ469211, GQ469090; *Epictia columbi* 4—GQ469214, GQ469092; *Epictia columbi* 5—GQ469215, GQ469093; *Epictia magnamaculata* 3—GQ469216, GQ469094; *Epictia phenops* 4—GQ469219, GQ469119; *Epictia phenops* 5—GQ469222, GQ469124; *Epictia phenops* 6—GQ469218, GQ469117). The leptotyphlopid snake *Siagonodon septemstriatus* (Schneider) was included as outgroup (GQ469232, GQ469116; Adalsteinsson et al., 2009). Methods used for the collection of the new DNA sequences are detailed elsewhere (Heinicke, Duellman & Hedges, 2007, Hedges, Duellman & Heinicke, 2008, Hedges & Conn, 2012). This work was approved by Penn State IACUC (41045).

Alignments were performed with (MUSCLE) in MEGA 6.06 (Tamura et al., 2013). The total concatenated alignment for the two genes was 1,269 aligned sites: 12S—471 sites, cytb—798 sites. A maximum likelihood analysis was performed using RAxML 8.0.9 (Stamatakis, 2014) through the CIPRES Science Gateway (Miller, Pfeiffer & Schwartz, 2010). The data were divided into four partitions (12S, cytb by codon position), and were analyzed using the evolutionary model GTRGAMMA, the maximized available option in RAxML. The same partitioning scheme was used for these genes in the study of Adalsteinsson et al. (2009), which involved sequences of the same species and where different partitioning schemes resulted in nearly identical trees. Gaps were treated as missing data. All parameters for the ML analyses were estimated by the program during the run. Branch support in the trees was provided by bootstrap analysis (2,000 replicates).

**Figure 1:**
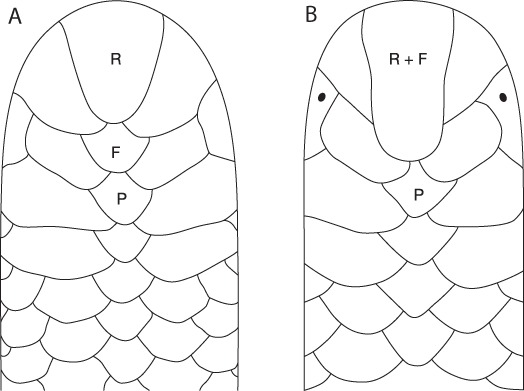
Dorsal head scale drawings of Epictia (modified from Taylor 1940) with dorsal head scales labeled following Wallach (2003). R, rostral; F, frontal (the prefrontal in all previous literature studying the taxonomy Middle American *E. goudotii* complex members); P, postfrontal. (A) rostral not fused with frontal scale; (B) rostral fused with frontal scale.

Authors of all literature mentioning dorsal head scales regarding *Epictia goudotii* and relatives from Mexico, Central America, and northern South America had called the dorsal head scale following the rostral scale a prefrontal scale, with that prefrontal scale fused with the rostral in some populations (i.e., Savage, 2002). That was until Pinto et al. (2010) called that prefrontal scale a frontal for their descriptions of *E. goudotii* and *E. magnamaculata* in Colombian territory. The source of the Pinto *et al.* different terminology was not cited, but came from Wallach (2003). Since the new terminology is likely to be confusing to workers familiar with the previous literature (all literature on *E. goudotii* complex except Pinto et al., 2010), we include an illustration of the head of two specimens with the new terminology illustrated (Fig. 1) and the old terminology explained. We use this new terminology knowing it will likely cause confusion among workers familiar with the literature on the Middle American segment of the *E. goudotii* complex in which the “old” head scale terminology is solidly entrenched and needed no change for that group.

Morphological variation among all leptotyphlopid snakes is summarized in Adalsteinsson et al. (2009). Abbreviations used here are: SVL = snout-vent length; TAL = tail length; and TOL = total length. TOL was measured by laying the snake alongside a standard 12 inch ruler containing mm (millimeters). All remaining measurements were made using dial calipers measured to the nearest 0.1 mm. Color statements given are those in alcohol unless otherwise noted. Because we are not aware of any external morphological characters to distinguish males from females, both sexes were combined for all morphological characters as was done for *E. goudotii* by Pinto et al. (2010). Also, Hedges (2008) found that New World leptotyphlopids are less sexually dimorphic than Old World species. The synonymies presented herein include the first use of any combination that pertains to the species in question, including all known misspellings, and the first reference to the single *i* and double *ii* spellings in various name combinations. Type numbers given for new species proposals and their stated type localities are also given for each new species proposal. Museum acronyms follow Sabaj Pérez (2014) and color names (capitalized) and numbered color codes (in parentheses) are those of Smithe (1975–1981).

In the following list of specimens examined, one asterisk denotes specimens from which external morphological data were taken whereas two asterisks designate specimens that were recorded only for the frontal scale condition. GenBank accession numbers are listed from (Adalsteinsson et al. (2009); in the order 12SrRNA, cytochrome b; n/a = gene not sequenced) for the old samples as well as the new genetic samples generated for this study. Two vouchers for genetic samples of Middle American specimens in Adalsteinsson et al. (2009) were not examined. ***Epictia ater.*** HONDURAS—CHOLUTECA: 3.2 km W of Choluteca, LSUMZ 10205* (see text); Choluteca, USNM 337534*; La Fortuna, UNAH 5286; 10–15 km W of San Francisco, LSUMZ 38815**. COMAYAGUA: 3 km W of Comayagua, TCWC 23815*; Comayagua, TCWC 23814*. CORTÉS: San Pedro Sula, LSUMZ 27741**, UNAH 0046, USNM 570405; 4 km E of Villanueva, UF 87867*–68*. EL PARAÍSO: El Rodeo, USNM 579865**. FRANCISCO MORAZÁN: Sabanagrande, UNAH 4723; Tegucigalpa, UNAH 2811. GRACIAS A DIOS: Tánsin, USNM 573213–21 (all *). INTIBUCÁ: near Jesús de Otoro, SMF 77788**; 5.5 km S of Jesús de Otoro, LSUMZ 33595**. LA PAZ: La Paz, UNAH 5344; Potrerillos, FMNH 283766**, 283741* (genetic sample 1: KP171710, KP171718), 283742*, USNM 581843* (genetic sample 2: KP171711, n/a). SANTA BÁRBARA: 10 km NNW of San José de Comayagua, UF 143892*; 14 km SSE of Santa Bárbara, UF 113592*. VALLE: Isla Conejo, USNM 580323**; Isla Inglesera, USNM 580322**; Isla Zacate Grande, KU 194335*, LSUMZ 36328**; near Nacaome, UF 113591*. YORO: 13.6 km NE of Jocón, UF 90018*; about 3 km S of San Lorenzo Arriba, USNM 565514*–15*; about 5 km SSE of San Lorenzo Arriba, USNM 578301**; about 4.7 km ESE of San Lorenzo Arriba, USNM 565516*–17*, 578302**–03**; San Patricio, USNM 579621–24 (all **). NICARAGUA—CARAZO: 20 km SW of Diriamba, SMF 81036*; GRANADA: Volcano Mombacho, SMF 78981*. MANAGUA: Managua, USNM 16134* (holotype of *E. nasalis*), 79957* (holotype of *E. ater*), 89479*. ***Epictia bakewelli***. MEXICO—GUERRERO: Chilpancingo, USNM 110305*; Ocotito, USNM 110307*. MICHOACÁN: La Salada, USNM 46340*; between Tecoman and Playa Azul, UTA R 54554* (see Materials and Methods; listed as *Epictia goudotii* 1 in Adalsteinsson et al., 2009). OAXACA: between Mazunte and Piontepanoc, UTA R-53657*; between Puerto Escondido and Puerto Angel, UTA R-57498* (see Materials and Methods; listed as *Epictia goudotii* 3 in Adalsteinsson et al., 2009). “No locality data,” FMNH 99676–79 (all *). ***Epictia goudotii*.** COLOMBIA—MAGDALENA: near Santa Marta, FMNH 165214** (scale data not taken because of small size). PANAMA—CANAL ZONE: “no other data,” FMNH 130672*, USNM 63110*. TRINIDAD—“no other data,” USNM 12498*. VENEZUELA—DISTRITO FEDERAL: “no other data,” USNM 121202–03 (both *). ***Epictia magnamaculata*.** HONDURAS—GRACIAS A DIOS: Islas del Cisne, Isla Grande, AMNH 36480**, UNAH 0982–83, 3616, USNM 142273*, 142361*, UNAH 256762*, 256774* (genetic sample 2: KP171714, KP171719); Islas del Cisne, Isla Pequeña, CAS 39405*–06*, MCZ R-9622–27 (all **). ISLAS DE LA BAHÍA: Cayo Cochino Grande, near La Ensenada, KU 220132*, UNAH 3637; Cayo Cochino Pequeña, UNAH 3643; Isla de Guanaja, East End, USNM 580324**; Isla de Guanaja, SE shore opposite Guanaja, CM 90324**, LACM 63428–32 (all*), UF 28580*; Isla de Guanaja, North East Bight, SMF 75998*; Isla de Guanaja, 2 km W of Savannah Bight, LSUMZ 21776–78 (all **); Isla de Guanaja, near Savannah Bight, SMF 78141**; Isla de Guanaja, Savannah Bight, SMF 78138–40 (all **), USNM 581844* (genetic sample 1: KP171713, n/a); “Isla de Guanaja,” CM 27618**, KU 101446*–47*, SMF 75999**; Isla de Roatán, Barbarette, UTA R-17019, 170120*–21**; Isla de Roatán, 0.5 km E of Corozal, USNM 563484*; Isla de Roatán, near Coxen Hole, FMNH 34593*; Isla de Roatán, between Flowers Bay and West End Point, USNM 565513*; Isla de Roatán, Mudd Hole Bay, SMF 80860*; Isla de Roatán, near Oak Ridge, UTA R-10717*–18*; Isla de Roatán, Palmetto Bay, FMNH 282651**; Isla de Roatán, Port Royal Harbor, LSUMZ 33774; Isla de Roatán, about 0.5 km N of Roatán, CM 90368*; Isla de Roatán, 0.5–1.0 km N of Roatán, LSUMZ 21775*; Isla de Roatán, 1.2 km E, 0.4 km S of Sandy Bay, KU 203163*; Isla de Roatán, Sandy Bay, KU 203164**–65*; “Isla de Roatán,” BMNH 1890.2.4.27–29; Isla de Utila, Iguana Station, SMF 79859**, 79868**, 81215**; Isla de Utila, 1 km NE of Utila, SMF 78001**; Isla de Utila, Utila, CM 90369*, UF 28399*–400*, 28438*–39*; “Isla de Utila,” AMNH 46425**, CM 29004**, LSUMZ 9702**, 22274*, 22296*, 22303*–04*, USNM 54760* (holotype); “no other data,” SMF 80889**. ***Epictia phenops*.** EL SALVADOR—EL SALVADOR: San Salvador, FMNH 154796, SMF 42022–23, 42025, 42801, 42933, 43216–18, 75814, 77236, 77399; Lago de Ilopango, SMF 42416. SAN VICENTE: km 40 on road towards San Vicente, SMF 77400; km 43 on road towards San Vicente, SMF 77400. SONSONATE: Hacienda San Antonio, SMF 42024. GUATEMALA—ALTA VERAPAZ: Cobán, USNM 6760 (paralectotype). HUEHUETENANGO: Nentón, UTA R-42208 (see Materials; listed as *Epictia goudotii* 4 in Adalsteinsson et al., 2009). HONDURAS—COPÁN: 1 km W of Copán, USNM 565518; Copán, UF 89459, USNM 563343; Santa Rita, UNAH 0716, 2474–75. LEMPIRA: 11.3 km NNW of Gracias, LSUMZ 23870. OCOTEPEQUE: Antigua, FMNH 283735* (genetic sample 2: KP171716, KP171721), 283736**, 283737* (genetic sample 1: KP171715, KP171720), 283738–40 (all **). MEXICO—CHIAPAS: Ocozocoautla de Espinosa, USNM 121465–67. OAXACA: Cajón de Piedra, USNM 110321; La Concepición, USNM 110320; Montaña Guengola, USNM 110319; Río Grande, USNM 110322; Tehuantepec, FMNH 111477, 111479, 111481, 111484, USNM 12444 (paralectotype), 30091–93, 30289 (paralectotype), 30290 (lectotype), 30291–95 (all paralectotypes), 30531–33, 46560, 110313–17. QUERÉTARO: Jalapán, USNM 46581. VERACRUZ: near La Victoria, UTA R-52658 (see Materials; listed as *goudotii* 6 in Adalsteinsson et al., 2009); Río Coatzacoalcos, USNM 61183; San Juan de la Punta, USNM 110308–11. YUCATÁN: Chichén Itzá, FMNH 20616, 36334–36, 36340; Dzibilchaltún, FMNH 153501, 153533, 153535–38, 153543, 153587; Kantunil, FMNH 36342; Mérida, FMNH 40724.

## Results

**Figure 2:**
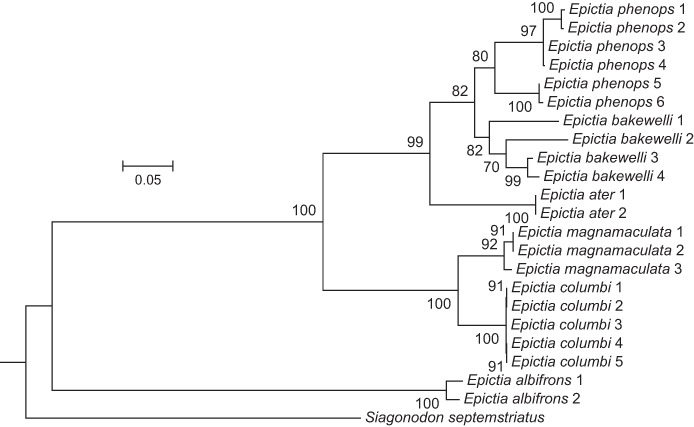
Phylogenetic tree of snakes of the genus *Epictia* from a maximum-likelihood analysis of DNA sequences of two mitochondrial genes (12S rRNA and cytochrome b). A scale bar showing sequence change is indicated. The numbers at nodes are bootstrap support values.

The molecular phylogeny (Fig. 2), which includes new sequence data and localities, supports the recognition of four taxa closely related to *E. goudotii* that have previously been described (Cope, 1875; Oliver, 1937; Taylor, 1940). Together with *E. goudotii*, these five species are supported as well by external morphological characters as described in the sections below (molecular data not available for Colombian *E. goudotii*, the country in which the type locality of *E. goudotii* occurs). As pointed out above, these five species were also recognized by Wallach, Williams & Boundy (2014), but those authors did not provide any evidence or comments to support their decisions.

The phylogenetic tree (Fig. 2) shows the same general pattern found by Adalsteinsson et al. (2009), namely, that *Epictia albifrons* is only distantly related to other species of the genus, the latter of which form two distinct clades. The Caribbean clade contains a pair of species (*E. columbi* and *E. magnamaculata*), whereas the Middle American Clade contains three species recognized here (*E. ater, E. bakewelli*, and *E. phenops*). One species in the Caribbean clade, *E. columbi*, is known only from a small Bahamian island (San Salvador) and thus it is not surprising that there is no genetic differentiation among those five individuals. However, it was unexpected to find, also, that there was little genetic difference among samples of *E. magnamaculata*, with localities separated by 280 km of open sea (Swan Islands and Bay Islands). The genetic structure within the Middle American clade is more pronounced (Fig. 2), showing deep divergences within *E. bakewelli* and *E. phenops*. Although these groups are also diagnosable morphologically, and are cohesive geographically (see below), the genetic differences within those two species are as great as that between *E. columbi* and *E. magnamaculata*, suggesting that there are additional species of *Epictia* in Middle America not yet recognized.

Based on the genetic results and the presence of a relatively broad dark dorsolateral stripe (see Peters & Orejas-Miranda, 1970), and following Adalsteinsson et al. (2009) & Cole et al. (2013; see their plate 36), who considered the Guyana specimens used herein for genetic data to represent *Epictia albifrons*, we likewise tentatively consider the two Guyana vouchers of the DNA data to be *E. albifrons* (but see Discussion). That species is apparently completely allopatric with *E. goudotii*, the latter, in our opinion (but too few genetic data and museum specimens available to us to do good analyses), occurring from coastal Venezuela, across northern Colombia, to the region of the Panamanian Canal Zone, and on Trinidad (see Materials and Methods). The remaining three mainland species treated herein (*E. ater, E. bakewelli*, and *E. phenops*) have a collective distribution from northwestern Costa Rica into Mexico as far north as coastal Jalisco on the Pacific versant and southern Tamaulipas, Mexico, on the Atlantic versant. Brief reviews of the synonymy, geographical distribution, diagnosis, variation, and remarks are provided for each of the five species of the *E. goudotii* complex studied herein. Neither *E. bakewelli* nor *E. phenops* have had good morphological descriptions published, but since all five species of the *E. goudotii* complex covered in this work are similar in overall external morphology, we only provide a detailed morphological redescription of *E. phenops*.

### *Epictia phenops (Cope)* (Fig. 3)

**Figure 3:**
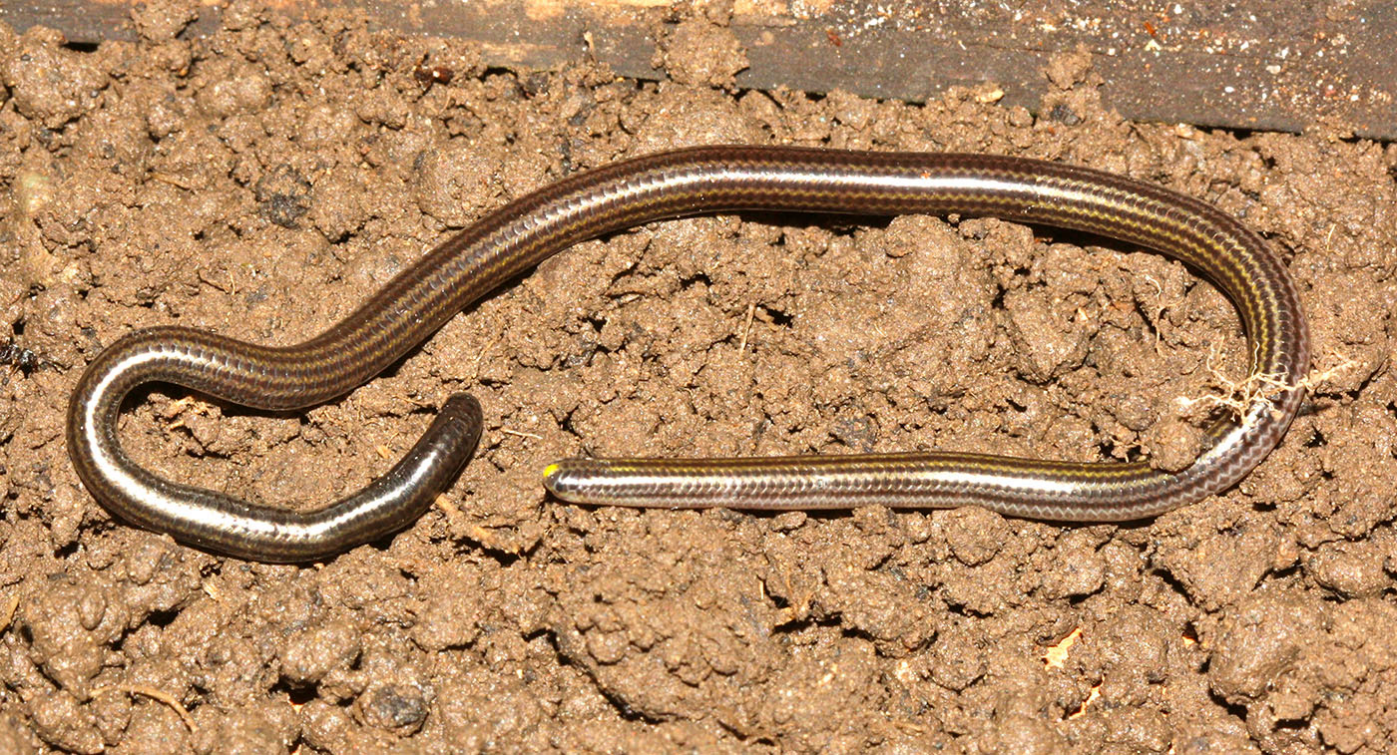
Adult of *Epictia phenops* (FMNH 283737) from Río Lempa, Ocotepeque, Honduras. Photograph by James R. McCranie.

*Stenostoma phenops* (Cope, 1875:128) (lectotype designated herein: USNM 30290, see Remarks): type locality: “Tehuantepec (Mexico)” and “Coban, Guatemala”; becoming “Santo Domingo Tehuantepec, Oaxaca, Mexico” by lectotype designation herein (also see Remarks).
*Stenostoma albifrons*: Bocourt, 1882:505 (in part), In: Duméril, Bocourt & Mocquard (1870–1909).
*Stenostoma dulce*: Bocourt, 1882:506 (in part), In: Duméril, Bocourt & Mocquard (1870–1909).
*Glauconia albifrons*: Boulenger (1893:63) (in part).
*Leptotyphlops albifrons*: Gaige (1936:298).
*Leptotyphlops bakewelli*: Oliver (1937:17) (in part).
*Leptotyphlops phenops*: Smith (1939:28).
*Leptotyphlops phenops phenops*: Smith (1943:445).
*Leptotyphlops ater phenops*: Dunn & Saxe (1950:159).
*Leptotyphlops goudotii phenops*: Peters & Orejas-Miranda (1970:170).
*Leptotyphlops goudotti phenops*: Pérez-Higareda & Smith (1991:28).
*Epictia goudotii*: Adalsteinsson et al. (2009:10) (in part).
*Epictia phenops*: Pinto et al. (2010:22) (in part).
*Epictia ater*: McCranie (2011:43) (in part).

**Figure 4:**
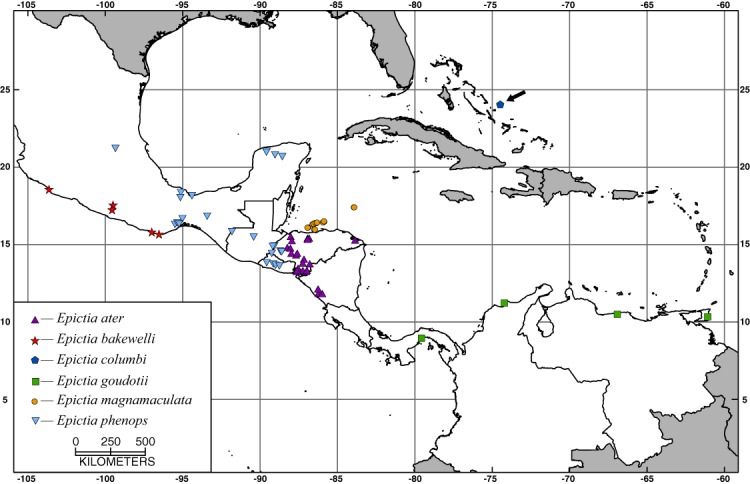
Map showing localities for specimens examined of the *Epictia goudotii* complex.

**Geographic distribution.** *Epictia phenops* occurs at low and moderate elevations from the relatively flat region of the Isthmus of Tehuantepec, Oaxaca, on the Pacific versant and southern Tamaulipas, Mexico, on the Atlantic versant southward to southwestern Honduras on the Pacific versant and extreme northern Belize and west-central Honduras on the Atlantic versant (Fig. 4; also see Remarks). See the Materials and Methods for a list of specimens examined and their locality data.

**Diagnosis.** *Epictia phenops* is one of the three species of the *E. goudotii* complex under study herein that lack rostral-frontal fusion (= has a frontal scale present). *Epictia ater* and *E. bakewelli* are distinguished from *E. phenops* in having rostral-frontal fusion. *Epictia goudotii* and *E. magnamaculata* are the two species that agree with *E. phenops* in having a frontal scale (= lack rostral-frontal fusion). *Epictia phenops* differs from *E. goudotii* in having the pale tail spot almost always larger ventrally than dorsally, covering 0–1 scales dorsally and 7–15 scales ventrally (versus pale tail spot, when present, larger dorsally than ventrally, covering 1–4 scales dorsally, 0–1 scales ventrally in *E. goudotii*). *Epictia phenops* differs from *E. magnamaculata* in usually having indistinct dark brown body stripes on a paler brown ground color, an indistinct to absent pale snout spot that is usually confined to the rostral when present, and a usually distinct pale tail spot that is almost always larger ventrally than dorsally (versus distinct alternating black and dark brown zig-zag body stripes, pale snout spot distinct with the spot almost always extending from the rostral onto adjacent edges of upper nasal scales, and a distinct pale tail spot that is almost always larger dorsally than ventrally, covering 2–6 scales dorsally and 2–5 scales ventrally in *E. magnamaculata*). The rare specimens (3 of 54 individuals) of *E. phenops* that have rostral-frontal fusion can be difficult to distinguish from *E. ater* and *E. bakewelli*, except that some *E. phenops* tend to have the anterior third of the venter paler brown than the adjacent dorsum (these surfaces usually about same color in *E. ater*).

**Description/Variation.** The following is based on 54 specimens examined (FMNH 153536; LSUMZ 23870; SMF 42022–25, 42081, 42933, 43216–17, 75813–14, 77236, 77399–400; UF 89459; USNM 6760, 12444, 30091–93, 30289–95, 30531–33, 46560, 46581, 61183, 110308–11, 110313–17, 110319–22, 121465–67, 563343, 565518; UTA R-42208, 52658; sexes not separated). *Epictia phenops* is a small threadsnake (maximum recorded TOL 179 mm (UTA R-52658)) with absent to distinct dark body stripes, dark stripes usually brown when present, stripes sometimes interrupted; with absent to distinct pale snout spot, pale snout spot almost always confined to rostral when present, extending onto adjacent edges of upper nasals in 1 of 54 individuals; pale tail spot usually distinct, larger ventrally than dorsally, covering 0–1 scales dorsally, 7–15 scales ventrally; under side of head and anterior third of body varies from distinctly paler brown than adjacent dorsum to only slightly paler brown than adjacent dorsum; body subcylindrical, head same width as neck; snout rounded in lateral and dorsal profiles; rostral reaching to level of eye when frontal present (51 of 54 individuals); rostral contacting supraoculars when fused with frontal (3 of 54 individuals), rostral not contacting supraoculars when frontal present (51 of 54 individuals); rostral width about 17–46 % of head width in 24; nostrils not visible from above; nasal divided, lower nasal much larger than upper nasal; upper nasals separated medially by rostral; frontal (when present) in contact with rostral, upper nasals, supraoculars, and postfrontal; supraoculars single, much larger than frontal; supraocular extending anteriorly to about level of mideye to just anterior to mid eye; supraocular not contacting anterior supralabial; frontal smaller than each supraocular; eye visible beneath scale, located anterior to center of ocular scale; supralabials 2; anterior supralabial extending dorsally only to level of lower third of eye; anterior supralabial in contact with lower and upper nasals and ocular scale; posterior supralabial in contact with ocular and parietal; infralabials 4–6, most often 5; mental present, divided; scales around body 14–14–14, all rows of equal size; median dorsal scales from rostral to tail spine 216–268 (240.3 ± 12.8, *n* = 54); medium ventral scales 196–249 (222.5 ± 13.4, *n* = 53); median subcaudal series 13–21 (17.3 ± 1.8, *n* = 54); scales around mid tail 10; enlarged precloacal scales absent; TOL 53–179 (131.7 ± 29.5, *n* = 54) mm; SVL 49–170 (122.4 ± 32.6, *n* = 54) mm; TAL 3.6–7.8 % of TOL in 54 individuals; diameter of midbody about 48–120 times into TOL in 54 individuals.

**Remarks.** Cope (1875:128) stated that the new species *Stenostoma phenops* “is represented by numerous specimens” with most having come from “Tehuantepec” and at least one was from “Coban, Guatemala.” Thus, the series on which Cope designated his new species became syntypes. Cochran (1961:214) listed nine USNM “Cotypes,” eight from Tehuantepec and one from Cobán, Guatemala. All nine specimens listed by Cochran still exist today in the USNM collection. Among those specimens, we designate USNM 30290 as the lectotype. The single measurement given by Cope most closely resembles USNM 6760 from Cobán, Guatemala. However, based on a review of the literature, we feel it is best to designate a specimen from Tehuantepec as the lectotype where eight of the nine “cotypes” listed by Cochran (1961) are from. USNM 30290 is an adult in good condition, with a TOL of 147 mm, lacks rostral-frontal fusion, has 244 median dorsal scales, 13 medial subcaudal scales, the pale snout spot confined to the rostral, the pale tail spot much larger ventrally than dorsally, thus agrees well with Cope’s description of the species. Thus, the remaining “cotypes” listed by Cochran (1961) become paralectotypes (USNM 6760, 12444, 30289, 30291–95) of *Stenostoma phenops* Cope. The type locality restrictions by Smith & Taylor (1945:24) & Smith & Taylor (1950:340) to “Tehuantepec” and “Tehuantepec (city, and environs),” respectively, were made without supporting data, thus are invalid.

Mertens (1952) wrote that one of the five specimens of *Epictia phenops* he examined from nearby localities in El Salvador had the rostral fused with the frontal scale (as praefrontale). Reexamination of those five specimens shows that one (SMF 43218) now lacks a head and the remaining four all have a frontal scale, although the suture between the rostral and frontal is indistinct in one (SMF 43216). Our specimens examined include all of the USNM specimens of *Epictia phenops* included by (Smith (1943), except for USNM 30094 (now soft and in bad condition), 110312 (exchanged), and 110318 (lost)), but with new scale counts in an effort to have a single person making those counts.

Pinto et al. (2010:22) suggested a species status for *Epictia phenops* for the populations “distributed from Mexico to Nicaragua,” thus that concept would be a composite of three species as recognized herein. Wallach, Williams & Boundy (2014) listed the Cozumel Island, Mexico, populations as both *E. magnamaculata* and *E. phenops*, but McCranie (2011) identified the Cozumel population as *E. magnamaculata*. Wallach, Williams & Boundy (2014) also recognized *E. phenops* as a species, but provided no data to support that decision. The concept of *E. phenops* of Pinto et al. (2010) cannot be the source for Wallach’s (1999, In: McDiarmid, Campbell and Touré) decision, because he recognized three species among the Pinto *et al. E. phenops*. Wallach, Williams & Boundy (2014) also included the El Salvadoran and western Honduran populations of *E. phenops* as *E. ater*.

Oliver (1937; as *Leptotyphlops albifrons* from Tehuantepec (=*Epictia phenops*)) included a drawing of the dorsal head scales of this species that shows the presence of the diagnostic frontal (= prefrontal of old terminology) scale, which should be helpful to the reader in visualizing that important character, especially considering the new head scale definition that will likely cause confusion since all previous workers on Middle American *E. goudotii* complex members have consistently used the old head scale terminology.

### *Epictia goudotii* (Duméril and Bibron)

*Stenostoma Goudotii* (Duméril & Bibron, 1844:330) (holotype: MNHN 1068, see Hahn, 1980:14); type locality: “vallée de la Magdeleine, à la Nouvelle-Grenade (= Colombia).”
*Stenostoma fallax* (Peters, 1857:402) (holotype: ZMB 9550, see Bauer, Günther & Klipfel, 1995:77); type locality: “Laguayra (= La Guajira, Venezuela).”
*Stenostoma Goudoti*: Jan (1861:188).
*Stenostoma albifrons*: Cope (1875:128).
*Stenostoma goudottii*: Cope (1875:129).
*Glauconia albifrons*: Boulenger (1893:63) (in part).
*Glauconia goudotii*: Boulenger (1893:64).
*Glauconia goudoti*: Werner (1917:203).
*Leptotyphlops goudotii*: Amaral (1930:139).
*Leptotyphlops goudoti*: Taylor (1940:540).
*Leptotyphlops albifrons*: Nicéforo Maria (1942:86).
*Leptotyphlops albifrons goudoti*: Dunn & Saxe (1950:159).
*Leptotyphlops albifrons albifrons*: Roze (1952:156) (in part).
*Leptotyphlops goudotii goudoti:*Peters & Orejas-Miranda (1970:169).
*Leptotyphlops goudotti goudotti*: Lancini (1979:170).
*Leptotyphlops goudotti*: Pérez-Santos & Moreno (1988:417).
*Leptotyphlops goudottii goudottii*: Pérez Santos (1999:295).
*Epictia goudotii*: Adalsteinsson et al. (2009:10) (in part).
*Epictia goudotti*: Ugueto & Rivas (2010:219).

**Geographic distribution.** *Epictia goudotii* is known to occur at low and moderate elevations from the Canal Zone, Panama, across northern Colombia and Venezuela, and on Trinidad (but see Remarks). See Materials and Methods for a list of specimens examined and their locality data (also see Fig. 4).

**Diagnosis.** *Epictia goudotii,* along with *E. magnamaculata* and *E. phenops*, are the three species of the *E. goudotii* complex under study herein that have a frontal (prefrontal) scale. *Epictia goudotii* differs from *E. magnamaculata* in having the pale snout spot very indistinct to distinct, with that spot usually confined to the rostral scale when distinct, and the pale tail spot nearly absent to indistinct (versus distinct pale snout spot almost always extending onto the adjacent edges of the upper nasal scales and the pale tail spot always distinct in *E. magnamaculata*). *Epictia goudotii* differs from *E. phenops* in having the pale tail spot nearly absent to indistinct, that spot almost always larger dorsally than ventrally, covering 1–4 scales dorsally and 0–1 scales ventrally (versus pale tail spot usually distinct and larger ventrally than dorsally, covering 0–1 scales dorsally and 7–15 scales ventrally in *E. phenops*). *Epictia goudotii* differs further from *E. ater* and *E. bakewelli*, the two species that lack a frontal (prefrontal) scale (= have rostral-frontal fusion) as follows: from *E. ater* in having the pale tail spot almost always larger dorsally than ventrally (versus tail spot, when present, much larger ventrally than dorsally in *E. ater*); from *E. bakewelli* in having the ventral surfaces about the same color as the dorsal surfaces (versus ventral surface of head and anterior third of venter distinctly paler than adjacent dorsum in *E. bakewelli*).

**Variation.** Pinto et al. (2010) gave the following morphological ranges for the *Epictia goudotii* specimens they examined: middorsal scales 227–260 (*n* = 7); midventral scales 213–234 (*n* = 5); subcaudal scales 12–16 (*n* = 7); TL 83–135 (*n* = 7) mm; TAL 4.4–6.6 (*n* = 7) % of TOL. Representative data for five specimens examined for this study (see Materials and Methods) are: middorsal scales 224–260 (242.3 ± 15.2, *n* = 4); midventral scales 212–246 (228.8 ± 14.4, *n* = 4); subcaudal scales 12–14 (13.2 ± 1.1, *n* = 5); TL 110–154 (133.6 ± 18.9, *n* = 5) mm; TAL 3.2–4.6 (*n* = 5) % of TOL. Other data for these five specimens include: SVL 105–148 (128.3 ± 18.4, *n* = 5) mm; dark body stripes indistinct to distinct, black to dark brown; frontal scale present in all 5; supraocular extending anteriorly to level of mideye, not contacting anterior supralabial; anterior supralabial extending dorsally only to level below eye to lower third of eye; pale snout spot very indistinct to distinct, spot usually confined to rostral (*n* = 4), extending onto adjacent edges of upper nasal in one; pale tail spot nearly absent to indistinct, larger dorsally than ventrally when visible, covering 1–4 scales dorsally and 0–1 scales ventrally; dark body stripes indistinct to distinct, black to dark brown.

**Remarks.** The erroneous spellings *goudottii* or *goudotti* started with Cope (1875) and continues to appear in some recent literature (*i.e.,* Savage, 2002; Ugueto & Rivas, 2010).

### *Epictia magnamaculata* (Taylor) (Fig. 5)

**Figure 5:**
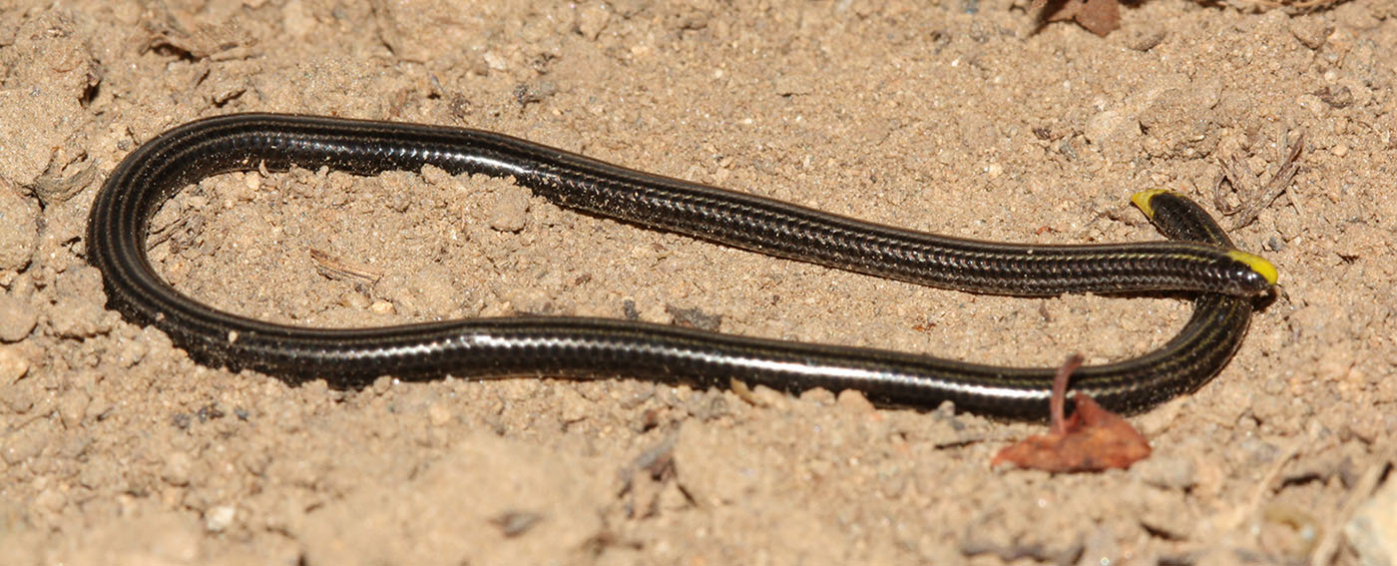
Adult of *Epictia magnamaculata* (FMNH 282651) from Palmetto Bay, Isla de Roatán, Islas de la Bahía, Honduras. Photograph by James R. McCranie.

*Glauconia albifrons*: Boulenger (1893:63) (in part).
*Leptotyphlops albifrons*: Barbour (1914:324).
*Leptotyphlops magnamaculata* (Taylor, 1940:532) (holotype: USNM 54760); type locality: “Utilla Id. (= Utila Island), Honduras.”
*Leptotyphlops albifrons magnamaculata*: Dunn & Saxe (1950:159).
*Leptotyphlops goudotii magnamaculatus*: Peters & Orejas-Miranda (1970:170).
*Leptotyphlops phenops*: Wilson & Hahn (1973:120) (in part).
*Leptotyphlops goudoti magnamaculata*: Schwartz & Thomas (1975:189).
*Leptotyphlops goudotii*: Wilson & Meyer (1982:23) (in part).
*Leptotyphlops goudotti*: Wilson & Meyer (1985:20) (in part).
*Epictia magnamaculata*: Adalsteinsson et al. (2009:10).

**Geographic distribution.** *Epictia magnamaculata* occurs at low elevations on the following Caribbean islands: the Bay Islands, including the Cayos Cochinos, Honduras; Islas del Cisne (Swan Islands), Honduras; Cozumel, Mexico; and San Andrés and Providencia, Colombia. See the Materials and Methods for a list of specimens examined and their locality data (also see Fig. 4).

**Diagnosis.** *Epictia magnamaculata*, along with *E. goudotii* and *E. phenops*, are the three species of the *E. goudotii* Species complex studied herein that have a frontal (prefrontal) scale. *Epictia magnamaculata* differs from *E. goudotii* in always having distinct black and brown body stripes on an overall black ground color, a distinct pale snout spot that almost always extends onto the adjacent edges of the upper nasal scales, and a distinct pale tail spot (versus pale snout spot very indistinct to distinct, spot usually confined to rostral, extending onto adjacent edges of upper nasal in one of five; pale tail spot nearly absent to indistinct in *E. goudotii*). *Epictia magnamaculata* differs from *E. phenops* in always having distinct black and brown body stripes on an overall black ground color, a distinct pale snout spot that almost always extends onto the adjacent edges of the upper nasal scales, and a distinct pale tail spot that is usually larger dorsally than ventrally (versus dark brown body stripes usually indistinct, pale snout spot very indistinct to distinct with the spot usually confined to rostral when distinct, and pale tail spot larger ventrally than dorsally in *E. phenops*). The rare *E. magnamaculata* specimen that lacks a frontal scale (3 of 58 individuals) differs from the two species that also lack a frontal (prefrontal) scale (*E. ater* and *E. phenops*) in having distinct black and brown body stripes, a distinct pale snout spot that almost always extends onto the adjacent edges of the upper nasal scales, and a distinct pale tail spot.

**Variation.** Pinto et al. (2010) gave the following morphological ranges for the specimens they examined from Islas de Providencia and San Andrés, Colombia: middorsal scales 245–262 (*n* = 12); midventral scales 237–246 (*n* = 4); subcaudal scales 15–18 (*n* = 13); TL 98–195 (*n* = 7) mm; TAL 4.8–7.1 (*n* = 12) % of TOL. Representative data for 35 additional specimens, including the holotype (see Materials and Methods), are: middorsal scales 216–244 (229.4 ± 8.6); midventral scales 198–227 (212.0 ± 9.0); subcaudal scales 15–20 (17.4 ± 1.5); TL 81–183 (133.5 ± 27.1) mm; TAL 4.5–8.8 % of TOL. Other data include: SVL 75–170 (124.8 ± 25.5) mm; frontal scale present in 55 of 58; supraocular extending anteriorly to about level of mideye to just anterior to mid eye; supraocular not contacting anterior supralabial; anterior supralabial extending dorsally only to level of lower third of eye; pale snout spot always distinct, spot almost always extending onto adjacent edges of upper nasal scales; pale tail spot always distinct, usually larger dorsally than ventrally, covering about 2–6 scales dorsally and 2–5 scales ventrally; alternating pale and dark zig-zag body lines distinct; ventral surfaces about same dark color as dorsum. Color in life of an adult (USNM 563484): “dorsal surfaces with Buff (24) and Jet Black (89) alternating zigzag stripes; top of rostral and adjacent scales and tail spot Spectrum Yellow (55)” (McCranie, 2011:484).

**Remarks.** The type description of *Leptotyphlops magnamaculata* provided by Taylor (1940) is fairly detailed and accurate, but based on only the holotype. Pinto et al. (2010) also provided information on morphological variation in a series (ca. 13) from the Colombian islands of Providencia and San Andrés and McCranie (2011) gave a detailed morphological description of this species (using the traditional head scale terminology of prefrontal as used in the Middle American *E. goudotii* complex) based on a series of 31 specimens from Honduran islands.

Taylor (1940) provided a head drawing of the holotype of *Epictia magnamaculata* showing the presence of the frontal (prefrontal) scale and the typical pale snout spot size. As evidenced from the non-overlap of some scale counts of *E. magnamaculata* from Islas de Providencia and San Andrés, Colombia versus specimens from the Bay Islands of Honduras, the former likely represents an undescribed species that we are currently studying.

Wallach, Williams & Boundy (2014:277) proposed *Leptotyphlops albifrons margaritae* Roze to be a synonym of *Epictia magnamaculata*, but without offering any supporting data. The description and illustrations of the head scales, along with the color description, of that nominal form provided by Roze (1952:154–156), however, are not convincing enough for us to agree that *E. a. margaritae* is a junior synonym of *E. magnamaculata*.

### *Epictia ater* (Taylor)

*Leptotyphlops* (= *Glauconia*) *albifrons*: Wettstein (1934:31).
*Leptotyphlops ater* (Taylor, 1940:536) (holotype: USNM 79947, see Wallach, Williams & Boundy, 2014:276); type locality: “Managua, Nicaragua.”
*Leptotyphlops nasalis*Taylor (1940:535) (holotype: USNM 16134); type locality: “Managua, Nicaragua.”
*Leptotyphlops albifrons*: Taylor (1951:27).
*Leptotyphlops albifrons ater*: Cochran (1961:194).
*Leptotyphlops phenops*: Campbell & Howell (1965:133).
*Leptotyphlops goudotii ater*: Peters & Orejas-Miranda (1970:170).
*Leptotyphlops goudotti*: Savage (1973:13).
*Leptotyphlops goudotii*: Savage (1980:16).
*Leptotyphlops goudotti ater*: Villa (1983:36).
*Leptotyphlops goudotti phenops*: Villa (1983:37).
*Epictia goudotii*: Adalsteinsson et al. (2009:10) (in part).
*Epictia nasalis*: Adalsteinsson et al. (2009:10).
*Epictia goudotii ater*: McConnell (2014:57).
*Epictia ater*: Wallach, Williams & Boundy (2014:275).

**Geographic distribution.** *Epictia ater* occurs at low and moderate elevations from western Honduras to northwestern Costa Rica, including Islas Murciélago (see Remarks). See the Materials and Methods for a list of specimens examined and their locality data (also see Fig. 4).

**Diagnosis.** *Epictia ater*, along with *E. bakewelli*, are the two species of this complex under study herein to have the rostral fused with the frontal (prefrontal) scale, thus the fused rostral-frontal scale contacts the postfrontal scale. *Epictia ater* differs most obviously from *E. bakewelli* in having the ventral surfaces essentially the same color as the dorsal surfaces (versus underside of head and about anterior third of venter distinctly paler brown than the brown dorsal surfaces in *E. bakewelli*). *Epictia ater* also differs from *E. bakewelli* in having the dark body stripes absent or indistinct (versus those stripes distinct in *E. bakewelli*). The single *E. ater* specimen with a frontal scale differs from two of the three species that also have a frontal scale, *E. goudotii* and *E. magnamaculata,* as follows: from *E. goudotii* in having the pale tail spot, when present, much larger ventrally than dorsally (versus tail spot larger dorsally than ventrally when present in *E. goudotii*); from *E. magnamaculata* in lacking distinct black body stripes and having the pale tail spot, when present, much larger ventrally than dorsally (versus distinct black stripes present and pale tail spot usually larger dorsally than ventrally in *E. magnamaculata*). A specimen of *E. ater* with a frontal scale can be difficult to distinguish morphologically from the normal *E. phenops*.

**Variation.** The following is based on 30 specimens examined, including the holotypes of *Epictia ater* and its synonym *E. nasalis* (see Materials and Methods): middorsal scales 212–259 (230.3 ± 11.7); midventral scales 192–242 (212.1 ± 11.7); subcaudal scales 15–21 (18.0 ± 1.7); TL 82–183 (129.3 ± 25.1) mm; SVL 78–174 (121.1 ± 23.9) mm; frontal absent in 40 of 41; supraocular extending anteriorly to about level of mideye to just anterior to mid eye; supraocular not contacting anterior supralabial; anterior supralabial extending dorsally only to level of lower third of eye; pale snout spot absent to distinct, confined to portion of rostral when present; pale tail spot absent to distinct, always much larger ventrally than dorsally when present, covering 0–2 scales dorsally and 4–12 scales ventrally; body stripes absent to indistinct; ventral surfaces essentially same color as dorsal color. Color in life of an adult (USNM 565514): “dorsum Jet Black (89), except for poorly distinguished slightly paler spot on rostral scale and pale yellow tail spine; venter Light Neutral Gray (85) anteriorly, grading to Jet Black posteriorly, except for pale yellow area on posterior portion of tail” (McCranie, 2011:44).

**Remarks.** The type descriptions of *Epictia ater* and its synonym *E. nasalis* provided by Taylor (1940) are fairly detailed and accurate. Unfortunately, external morphological variation in the species was poorly documented until McCranie (2011) provided a detailed description of *E. ater* based on Honduran specimens. However, the McCranie (2011) description also included data from four specimens (of 28 included) now known to represent *E. phenops*.

No museum specimens of *Epictia ater* from Costa Rica were available to us at the time we were borrowing museum specimens. However, Savage (2002:559) stated the *E*. (as *Leptotyphlops*) *ater* from Costa Rica were in agreement with the *E. ater* of Nicaragua (including the holotype) in having “rostral-prefrontal fusion” (= rostral-frontal fusion) and were also in agreement with the color pattern of the Nicaraguan specimens. For those reasons, Savage (2002) elevated *E. ater* to a valid species for the Nicaragua and Costa Rica populations. As noted in McCranie (2011) and in this work, most Honduran populations agree with those characters and are assigned with the Nicaragua and Costa Rica populations to the species *E. ater*. Thus, without our examination of Costa Rica specimens, we are confident in assigning Costa Rican specimens to *E. ater* along with those of Nicaragua and Honduras. The range of *E. ater* terminates in northwestern and north-central Costa Rica.

Savage (2002) gave USNM 79947 for both holotypes of *Epictia ater* and its synonym *E. nasalis*, probably because Taylor gave the correct USNM number (79947) in the legend for his Fig. 4, but gave an incorrect number in his list of the designation of the holotype. Taylor (1940) included head drawings of *E. ater*, and its synonym *E. nasalis*, showing the absence of the frontal scale. Wallach, Williams & Boundy (2014) included the El Salvadoran and western Honduran populations as *E. ater* (in error). However, the molecular and external morphological data for those populations from El Salvador and Copán, Honduras, suggest they are *E. phenops*. Because of the overall similarity in dorsal color pattern of *E. ater* with that of *E. phenops*, no photograph of *E. ater* is included herein.

### *Epictia bakewelli* (Oliver)

*Leptotyphlops bakewelli* (Oliver, 1937:16) (holotype: UMMZ 80228); type locality: “Paso del Río, Colima, Mexico.”
*Leptotyphlops phenops bakewelli*: Smith (1943:445).
*Leptotyphlops ater bakewelli*: Dunn & Saxe (1950:160).
*Leptotyphlops gadowi* (Duellman, 1956:93) (holotype: BMNH 1946.9.7.55, see McDiarmid, Campbell & Touré 1999:31): type locality: “above Apatzingan,” Michoacán, Mexico.
*Leptotyphlops goudotii bakewelli*: Peters & Orejas-Miranda (1970:170).
*Leptotyphlops goudotii phenops*: Hahn (1980:15) (in part).
*Leptotyphlops goudotti bakewelli*: Pérez-Higareda & Smith (1991:29).
*Epictia bakewelli*: Wallach, Williams & Boundy (2014:276).

**Geographic distribution.** *Epictia bakewelli* occurs at low and moderate elevations from Colima to the foothills west of the Isthmus of Tehuantepec, Oaxaca, Mexico. See the Materials and Methods for a list of specimens examined and their locality data (also see Fig. 4).

**Diagnosis.** *Epictia bakewelli*, along with *E. ater*, are the two species of this complex to have the rostral fused with the frontal (prefrontal) scale, thus the fused rostral-frontal scale contacts the postfrontal scale. *Epictia bakewelli* differs most obviously from *E. ater* in having the underside of head and about the anterior third of the venter distinctly paler brown than the adjacent brown dorsal surfaces (versus ventral surfaces essentially the same color as the dorsal surfaces in *E. ater*). *Epictia bakewelli* also differs from *E. ater* in having distinct dark body stripes (versus those stripes indistinct or absent in *E. ater*). *Epictia bakewelli* differs further from two of the three species that have a frontal scale (*E. goudotii* and *E. magnamaculata*) in having the underside of head and about anterior third of the venter distinctly paler brown than the adjacent brown dorsal surfaces and the pale tail spot larger ventrally than dorsally (versus those ventral surfaces similar to dorsal surfaces and the pale tail spot larger dorsally than ventrally in *E. goudotii* and *E. magnamaculata*). The rare specimen of *E. phenops* that lacks a frontal scale (3 of 54) can be difficult to distinguish from *E. bakewelli*.

**Variation.** Our data from *Epictia bakewelli* are based on only ten specimens examined (see Materials and Methods) plus limited data extracted from the Oliver (1937) & Duellman (1956) descriptions of the holotypes of *E. bakewelli* and its synonym *E. gadowi*, respectively: middorsal scales 226–262 (242.1 ± 10.7, *n* = 12); midventral scales 206–244 (223.0 ± 11.1, *n* = 11); subcaudal scales 14–20 (17.9 ± 2.1, *n* = 11); TL 84–145 (116.4 ± 21.4, *n* = 11) mm; SVL 78–135 (108.8 ± 20.3, *n* = 11) mm; TL 4.7–8.0 (*n* = 11) % of TOL; frontal absent in all 12; supraocular extending anteriorly to about level of mideye to just anterior to mid eye; supraocular not contacting anterior supralabial; anterior supralabial extending dorsally only to level of lower third of eye; pale snout spot absent to distinct, usually confined to rostral when present (extending onto adjacent edges of upper nasal in two of ten); pale tail spot distinct, larger ventrally than dorsally, covering 0–4 scales dorsally (confined to tail spine in two of ten), and 1–11 scales ventrally; dark stripes on body distinct in all, zig-zag shaped; under surface of head and at least adjacent third of belly much paler brown than adjacent dorsum.

**Remarks.** The type descriptions of *Epictia bakewelli* and its synonym *E. gadowi* (both as *Leptotyphlops*) were brief and lacked much standard scale data and an adequate study of the variation in scales in *E. bakewelli* has not been published.

Oliver (1937) & Duellman (1956) provided dorsal head drawings of the holotypes of *Epictia bakewelli* and *E. gadowi* (a synonym of *E. bakewelli*), respectively. Those drawings show the absence of the frontal scale, one of the diagnostic characters of this species. Wilson & Hahn (1973) suggested placing *E. bakewelli* in the synonymy of *E. phenops* based on variation in the presence or absence of rostral-frontal fusion (= presence or absence of a frontal scale), but that action was based on little evidence and also ignored the detailed color pattern of *E. bakewelli* given by Oliver (1937). The color pattern, along with the absence of the frontal scale are the two most important morphological diagnostic characters to distinguish *E. bakewelli* from the remaining members of the *E. goudotii* Species complex. No quality photographs of *E. bakewelli* were available to us to include herein.

One of the *Epictia bakewelli* specimens examined by Smith (1943; USNM 110306) has been subsequently lost and another (USNM 30295) identified as *Leptotyphlops phenops bakewelli* by Smith (1943) has been reidentified as *E. phenops*. Recently, Wallach, Williams & Boundy (2014:276) recognized *E. bakewelli* as a species, but provided no data to support that decision.

## Discussion

Here, we revise the *Epictia goudotii* Species complex to include six species: *E. ater, E. bakewelli, E. columbi* (not studied morphologically herein)*, E. goudotii, E. magnamaculata*, and *E. phenops*. Adalsteinsson et al. (2009) showed with molecular data that *E. albifrons* is not closely related to these species and is therefore not a member of the *Epictia goudotii* Species complex, even though it has been confused with *E. goudotii* in the past. Therefore, *Epictia albifrons*/*E. tenella* and relatives should be considered a separate complex, the *Epictia albifrons* Species complex. Although we lacked genetic data for *E. goudotii*, we consider it to occur from the Panama Canal Zone to northern Colombia and Venezuela (see also Pinto et al., 2010).

Molecular clock estimates of divergence times (Adalsteinsson et al., 2009) indicate that the *Epictia goudotii* Species complex split from *E. albifrons* in the early Cenozoic and that the Caribbean and Middle American Clades of the complex diverged from one another in the Oligocene (∼30 million years ago). Even closely related species, such as *E. columbi* and *E. magnamaculata*, appear to have diverged more than 10 million years ago (Adalsteinsson et al., 2009). These deep splits provide further support for the recognition of species, rather than subspecies, in this Species complex, and suggest that additional species remain to be recognized given the phylogenetic structure within *E. bakewelli* and *E. phenops*. The presence of *E. columbi* in the Bahamas, in itself, indicates that these snakes can disperse over ocean waters. Additional support for oceanic dispersal comes from this deep timeline (Adalsteinsson et al., 2009), which infers that Middle America was invaded by these snakes (from South America) prior to the emergence of the Isthmus of Panama approximately 3 million years ago.

As noted in the introduction, the taxonomic history of the *Epictia goudotii* complex has been complicated and confused. *Epictia albifrons* and *E. tenella* lie outside the scope of this work, and because they are not closely related to the *E. goudotii* complex (Adalsteinsson et al., 2009), are discussed herein only because we used sequences tentatively identified as *E. albifrons*/*E. tenella* in our molecular phylogeny, as an outgroup. *Epictia albifrons* has also been confused with *E. goudotii* in some earlier literature. Some debate occurs in the literature about the validity of one or both of those nominal forms. Both have sometimes been recognized as valid species (Wallach, Williams & Boundy, 2014; but without documentation), or *E. albifrons* was considered a *nomen dubium* (Franco & Pinto, 2010), or *E. tenella* was considered a junior synonym of *E. albifrons* (Hoogmoed & Gruber, 1983). Klauber (1939:59) diagnosed *E. tenella* from *E. albifrons* by the “contact between supraoculars and the anterior supralabial” in *E. tenella* versus the absence of that contact in *E. albifrons* because “the junction of the nasal and ocular” prevented such contact. The destroyed holotype of *E. albifrons* (but see neotype designation in Mumaw, González & Fernández, 2015) along with the poor description of the holotype and the possibly erroneous type locality data all contribute to this problem.

Orejas-Miranda (1967) considered both *Epictia albifrons* and *E. tenella* to have large geographical distributions that included Guyana. Orejas-Miranda (1967) noted the absence versus presence of the supraocular-anterior supralabial contact in those two species, but could not find any museum specimens representing, in his opinion, *E. albifrons*. Peters & Orejas-Miranda (1970) recognized both *E. albifrons* and *E. tenella* as valid species, and also noted the presence (*E. tenella*) or absence (*E. albifrons*) of the supraocular-anterior supralabial contact, but included only *E. tenella* in the fauna of the “Guianas.”

Hoogmoed (1977) studied the threadsnakes from Suriname and assigned the name *Epictia* (as *Leptotyphlops*) *tenella* to those populations. Hoogmoed & Gruber (1983) suggested placing *E. tenella* in the synonymy of *E. albifrons* in order to provide a much-needed diagnosis of *E. albifrons*. Hoogmoed & Gruber claimed that a combination of characters they could see in Wagler’s (1824) illustration of *Stenostoma albifrons* strengthened their opinion, but we think that Wagler’s illustration is too poor to be of any use in species identification and the Hoogmoed & Gruber (1983) decision to place *E. tenella* as a junior synonym of *E. albifrons* is premature. Klauber (1939:61), regarding the supraocular-anterior supralabial contact, stated, “The available specimens indicate that in this area (Guyana and Trinidad) the character is quite consistent, although in one specimen (Carnegie Museum 4890) these scales fail to make contact on the right side of the head.” Klauber (1939:61) also said, “Among the specimens of the *albifrons*-group which I have had available from South and Central America these from British Guiana and Trinidad have been the only ones having the anterior supralabials in contact with the supraoculars.” Klauber (1939) recorded *E. tenella* from Trinidad (with AL-SO contact), but the single Trinidad specimen examined for the present work (USNM 12498) lacks that contact. Considering that we found almost no variation, and what occurred was minor, in the sizes and extent of the anterior supralabial and supraocular in the large series of *Epictia* we examined (except the aberrant absence of the supraocular in the holotype of *E. nasalis*; but see Dunn & Saxe (1950) who noted variation in this character in 2 of 5 specimens from Nicaragua that were not examined by us), we doubt that significant variation occurs in which the supraocular can regularly extend anteriorly to contact the anterior supralabial in one specimen but not in another of the same species. We agree more with the statement by Thomas (1965:6) that “There is obviously more than one species involved in the material (of *E. albifrons*) I have seen” when he was discussing “South American mainland” specimens he examined. Recently, Mumaw, González & Fernández (2015) considered *E. tenella* to represent a Species complex. Clearly, a combined DNA and morphological study of South American populations of this complex is needed, especially one that uses both types of data sets with northern South American populations that contain both the anterior supralabial-supraocular contact and those that lack that contact. However, current export prohibition of tissues from several South American countries is a serious obstacle to such a study. Also, it is unfortunate that Pinto et al. (2010) did not study genetic data in their review of Colombian Epictini as Colombian tissues are not available for export. Franco & Pinto (2010) also did not include genetic data in their work on the *E. albifrons*/*E. tenella* problem.

### Key to the species of the *E. goudotii* Species complex

1
  A. Frontal (prefrontal) scale usually absent……………2
  B. Frontal (prefrontal) scale usually present……………3
2
  A. Anterior third of venter essentially same color as adjacent dorsum; distinct dark dorsal and lateral body stripes absent…………*E. ater*
  B. Anterior third of venter distinctly paler brown than adjacent dorsum; distinct dark dorsal and lateral body stripes present………*E. bakewelli*
3
  A. Fewer than 22 subcaudal scales……………4
  B. 22 or more subcaudal scales………*E. columbi*
4
  A. Pale snout spot, when present, almost always confined to rostral………5
  B. Pale snout spot almost always involves at least parts of supranasal and frontal, as well as rostral………*E. magnamaculata*
5
  A. Pale area on tail almost always larger dorsally than ventrally………*E. goudotii*
  B. Pale area on tail almost always larger ventrally than dorsally………*E. phenops*

## Additional Information and Declarations

### DNA Deposition

The following information was supplied regarding the deposition of DNA sequences: Genbank Accession numbers are provided in text.

### Data Deposition

The following information was supplied regarding data availability: No additional raw data beyond the Genbank accession numbers found in the body of the manuscript.
